# An integrated multi-electrode-optrode array for *in vitro* optogenetics

**DOI:** 10.1038/srep20353

**Published:** 2016-02-02

**Authors:** Marleen Welkenhuysen, Luis Hoffman, Zhengxiang Luo, Anabel De Proft, Chris Van den Haute, Veerle Baekelandt, Zeger Debyser, Georges Gielen, Robert Puers, Dries Braeken

**Affiliations:** 1 Imec, Life Science Technologies Department, Kapeldreef 75, Heverlee, 3001, Belgium; 2 KULeuven, Engineering Department, Kasteelpark Arenberg 10 - bus 2443, Leuven, 3000, Belgium; 3 KULeuven, Neurobiology and Gene Therapy, Kapucijnenvoer 33 blok i - box 7001, Leuven, 3000, Belgium; 4 KULeuven, Leuven Viral Vector Core, Leuven, 3000 Belgium; 5 KULeuven, Molecular Virology and Gene Therapy, Kapucijnenvoer 33 blok i - box 7001, Leuven, 3000, Flanders, Belgium

## Abstract

Modulation of a group of cells or tissue needs to be very precise in order to exercise effective control over the cell population under investigation. Optogenetic tools have already demonstrated to be of great value in the study of neuronal circuits and in neuromodulation. Ideally, they should permit very accurate resolution, preferably down to the single cell level. Further, to address a spatially distributed sample, independently addressable multiple optical outputs should be present. In current techniques, at least one of these requirements is not fulfilled. In addition to this, it is interesting to directly monitor feedback of the modulation by electrical registration of the activity of the stimulated cells. Here, we present the fabrication and characterization of a fully integrated silicon-based multi-electrode-optrode array (MEOA) for *in vitro* optogenetics. We demonstrate that this device allows for artifact-free electrical recording. Moreover, the MEOA was used to reliably elicit spiking activity from ChR2-transduced neurons. Thanks to the single cell resolution stimulation capability, we could determine spatial and temporal activation patterns and spike latencies of the neuronal network. This integrated approach to multi-site combined optical stimulation and electrical recording significantly advances today’s tool set for neuroscientists in their search to unravel neuronal network dynamics.

Neuromodulation opens perspectives for the treatment of an increasing number of neurological diseases. Nowadays, modulation by electrical stimulation is already used in the clinic by neurosurgeons to treat chronic pain, movement disorders and psychiatric illnesses (e.g. Parkinson’s disease^1^ and obsessive-compulsive disorder^2^). However, electrical neuromodulation is hampered by a lack of specificity, which severely impacts its effectiveness and safety^1^. Ideally, neuromodulation should allow precise targeting of specific cell populations, resulting in some form of modulation, e.g. activation or inhibition of targeted cells. Effective modulation of nerve cells also requires stimulation with millisecond precision and enable cross talk-free simultaneous monitoring of electrical activity, thereby enabling closed-loop therapies.

Optogenetics is a new neuromodulation technique which allows neurons to be controlled by light, instead of electrical current^3^,^4^. For this purpose, neurons of interest are genetically encoded to produce and express light-sensitive proteins, like channelrhodopsin^5^ (ChR2) and halorhodopsin^6^. Once these proteins are expressed, neural activity can be regulated by exposing the cells to light of the appropriate wavelength. In this way, optogenetics provides multi-modal control over neural function, genetic targeting of specific cell types, and the potential to reduce electrical stimulation artifacts while recording electrical activity. Together, these versatile features combine to a powerful tool set for the study of neural circuitry and treatment of psychiatric and neurological disorders.

The advent of optogenetics was followed by an extensive development of new technologies that enable distribution of light inside the brain, or that combine optogenetics with other modalities including electrophysiology^7^,^8^,^9^,^10^,^11^. However, most current optical devices have limited capabilities or require complicated, bulky setups. Essential problems in current devices are tissue temperature increase due to excessive power consumption, impractical connections to external light sources and limited or no spatial addressability due to the use of single fibers^12^,^13^. An example of an optical-electrical device was reported in which one electrode on Utah-style probe arrays was replaced by an optical fiber^10^. The drawback of this approach is that the number of optical fibers would need to increase to accommodate more optical outputs and that it requires a cumbersome manual assembly process. In another example, Michigan-style probes were fabricated using a modified process that incorporated SU-8 waveguides to transport light into the brain^14^. Although the monolithic fabrication is an improvement compared to the use of bulk optic fibers, the waveguide cross section area is relatively large (about 15 × 5 μm^2^) and light is still delivered to the waveguide through an optical fiber which is glued onto the waveguide. Thirdly, another type of device has been developed that incorporates light emitting diodes (LEDs)^15^,^16^,^17^. Although this approach avoids the use of optical connections, the LEDs are either not monolithically integrated with the substrate, or they require a complex optical system to project the light onto the sample. Furthermore, the smallest known LED solution for optogenetics^17^ is still bulky when compared to normal electrical only probes and it requires discrete assembly, which is more complex than monolithically integrated solutions. Also, since the LEDs are in close contact to the tissue, an undesirable temperature increase is more difficult to avoid. Another common problem arises when optical stimulation and simultaneous electrical recording are combined, i.e. the light stimulus induces electrical artifacts which disturb the recording of the neuronal response^18^. These can be caused directly by the photovoltaic effect or indirectly by increasing the electrode temperature, which changes the electrical properties of the material. This poses a challenge for registration of electrical activity immediately after the stimulation pulse or during high frequency stimulation protocols, *in vitro*^19^ as well as *in vivo*^20^.

To address these challenges, we fabricated a novel 8 × 8 multi-electrode-optrode array (MEOA) for *in vitro* optogenetic applications, in which we monolithically integrated titanium nitride (TiN) electrodes with silicon nitride (SiN) waveguides. We present here the design, fabrication and characterization of the device, and demonstrate the capability to reliably elicit spikes and change baseline activity of the neuronal network growing atop the MEOA. Moreover, we show that, thanks to the small size of the blue optrodes (6 × 23μm), single neuron stimulation is possible and therefore the MEOA can be used to spatially and temporally characterize network activation patterns and spike latencies.

## Results

### System design and characterization of the MEOA

**Figure 1:**
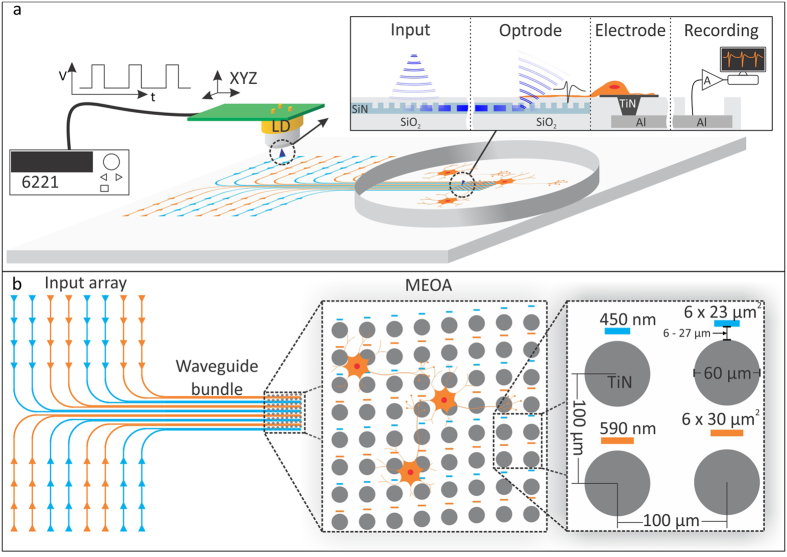
Conceptual drawing of the stimulation/recording setup and layout of the multi-electrode-optrode array (MEOA) chip. (**a**) The light is introduced into the system at the input array region, which is located at the edge of the chip. There is a grating coupler at each location (indicated with a triangle) that couples light into a particular waveguide when a light source is placed above it. The light is then carried by the corresponding waveguide into one of the optrodes (output grating coupler). (**b**) The MEOA is composed by an array of eight by eight optrodes and TiN electrodes. There are two types of optrodes for two different wavelengths (450 nm, corresponding to blue, and 590 nm, corresponding to amber), which are vertically interlaced. The electrode diameter is 60 μm and the optrodes are 6 × 23 μm^2^ and 6 × 30 μm^2^ for blue and amber, respectively. The pitch is 100 μm in both directions for the electrodes and 200 μm vertically by 100 μm horizontally for each type of optrode.

The MEOA chip was designed as a combination of a conventional multi-electrode array (MEA) supplemented with optical outputs (optrodes) positioned in between and close to the electrodes (see Fig. 1). The optical stimulation array consists of 64 optrodes in a rectangular 8 by 8 configuration. Half of the optrodes are designed to output light of 450 nm (blue) and the other half light of 590 nm (amber). For each output of the MEOA, the light is carried by a single waveguide from a corresponding location in the input array region. In order to activate a particular output a light source is placed on top of its corresponding input (see Fig. 1a).

All inputs and outputs are formed by optical grating couplers that introduce or extract light into or out of the SiN waveguides (see Fig. 1a inset). This photonic platform presents several advantages: first, it allows miniaturizing the light outputs (optrodes) down to sizes comparable to a cell body; second, the light is projected under an angle of 20° to the normal towards the cells; and third, the waveguides are fabricated at wafer level and are fully integrated with the electrode array, which is a reliable, reproducible and scalable process.

The grating couplers were designed and optimized using the methodology reported in^21^. The optrode sizes are 6 × 23 μm^2^ and 6 × 30 μm^2^ for the blue and amber outputs, respectively. These sizes are comparable to a neuronal cell body, therefore allowing modulation of neuronal networks with single cell precision.

The two different kinds of optrodes are vertically interlaced, rendering a vertical pitch of 200 μm and horizontal pitch of 100 μm for each type (see Fig. 1b). There is an electrode of 60 μm in diameter next to each optrode, forming an array of 64 electrodes with a pitch of 100 μm in each direction. The distance between the electrodes and the optrodes is kept small (ranging from 6 to 27 μm) to allow recording the response of a cell that is stimulated near the electrode.

**Figure 2:**
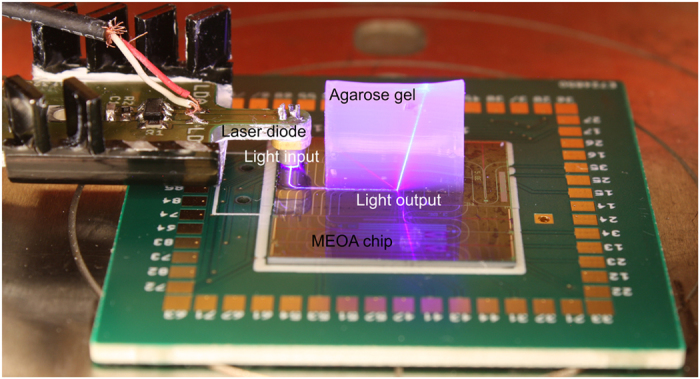
Photograph of the MEOA setup with light input, waveguides and output (optrode) visible. The output light beam was made visible by placing a block of 3% agarose gel on top of the optrode.

We chose to characterize the MEOA using blue light to be able to use the very common ChR2 opsin. First, we determined if the light intensity delivered by the blue optrodes was sufficient to elicit action potentials in ChR2-expressing neurons. We therefore aligned a blue diode laser above the input grating and measured the light power at the output grating. Figure 2 shows the MEOA setup with light input, waveguides and output (optrode) visible. The output light beam was made visible by placing a block of 3% agarose gel on top of the optrode. The total output power measured in 32 optrodes was 12 ± 0.71 μW, recalculated to a light output density of 87 mW/mm^2^ (output area of 6 × 23 μm^2^), which is well above the known threshold for generating action potentials in transduced neurons^22^. The low-impedance TiN electrodes embedded on the MEOA provide a stable and reliable recording interface. The impedance of the 60 μm diameter contacts was 10.65 ± 1.17 kOhm (mean ± SD). These values did not significantly change even after 5 times washing, coating and plating of new cells on the MEOA’s surface (Wilcoxon matched pairs test, p = 0.305, n = 182 electrodes measured from 5 MEOA’s).

### Biocompatibility and electrical performance of the MEOA

**Figure 3:**
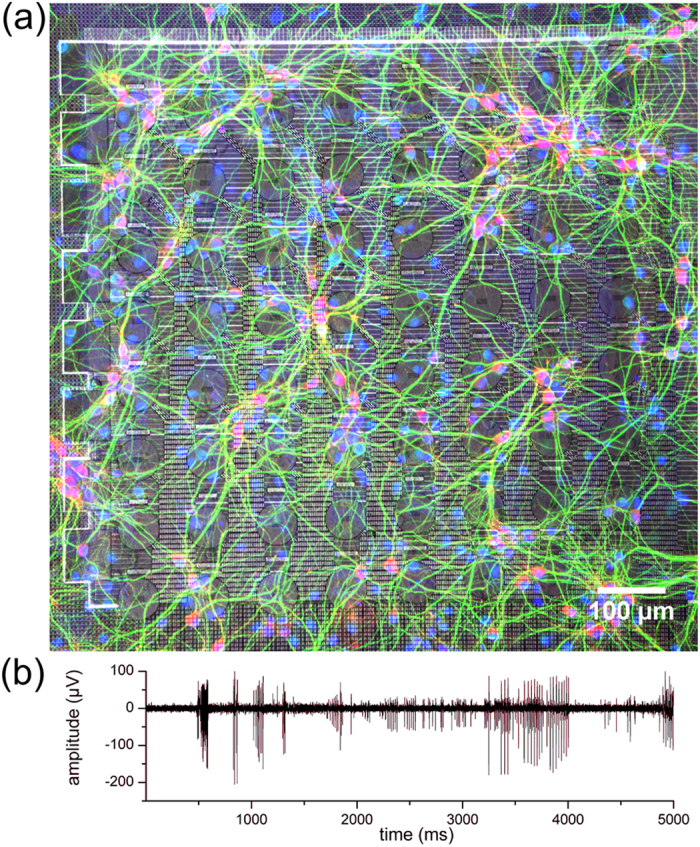
Demonstration of biocompatibility and recording capabilities of the MEOA chip. (**a**) Confocal fluorescence image of a transduced hippocampal neuronal network (DIV15) cultured on the MEOA. Transduced neurons are shown in red (mCherry), neuronal dendrites in green (Anti-MAP2) and cell nuclei in blue (DAPI). Transduction rate was on average 51.8 ± 13.4% (n = 5 cultures). Brightness and contrast are enhanced for visualization purposes. (**b**) Representative baseline recording of hippocampal neurons on one electrode contact at DIV13 with an SNR of >10.

The MEOA was fabricated in the imec cleanroom using a fully biocompatible CMOS process. To illustrate the excellent biocompatibility of the chip fabrication and packaging, primary hippocampal neurons were cultured on top of the chip. They formed stable networks that were viable for at least 3 weeks. Figure 3a depicts a representative neuronal culture on the MEOA showing the ChR2 opsin expression in transduced neurons (red), neuronal dendrites (green), and cell nuclei (blue) at day *in vitro* (DIV) 15. Figure 3b shows signals from spontaneously active neurons on an electrode at DIV13 with signal amplitudes up to 341 μV (peak to peak) and a signal to noise ratio larger than 10. The baseline noise level was very low and mainly dependent on the noise added by the commercial MEA amplifier: 1.95 ± 0.15 μV_RMS_ (mean ± SD, 317 contacts on 5 MEA’s) for recordings with the USB-ME32-FAI-System and 1.49 ± 0.09 μV_RMS_ (mean ± SD, 286 contacts on 5 MEA’s) for the MEA1060-Inv-BC system.

### Combined electrical recording and optical stimulation

A common problem when combining optical stimulation with simultaneous electrical recording is that the light stimulus can induce electrical artifacts^18^. TiN films naturally form a titanium dioxide (TiO_2_) layer when exposed to air or an electrolyte^23^. Being a semiconductor, TiO_2_ presents photovoltaic effects known to these types of materials when placed in a liquid^24^. For this reason, one would expect the presented array to be susceptible to these recording artifacts. This would thus interfere with the extracellular recording of single cell activity.

**Figure 4:**
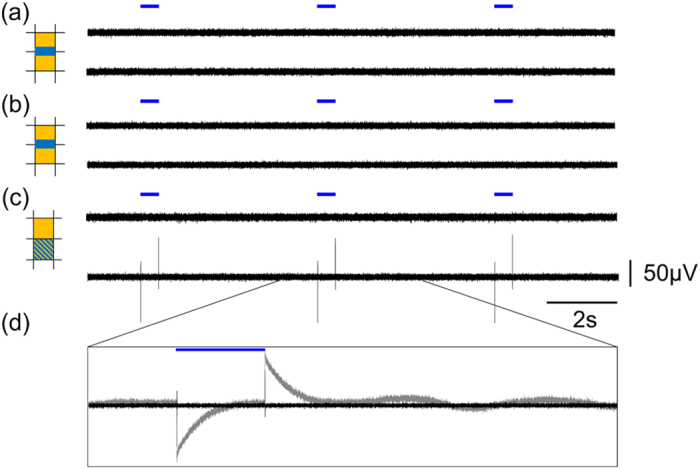
Demonstration of the absence of the photo-induced light artifact in the MEOA. Bandpass filtered signal recorded in neurobasal medium of 2 neighboring electrodes (indicated on the left in orange) when stimulating through the indicated optrode (indicated on the left in blue) with a pulse width of 500 ms. (**a**) Output intensity of 5 μW. (**b**) Output intensity of 120 μW. (**c**) Bandpass filtered signal recorded in neurobasal medium of 2 neighboring electrodes (in orange) when stimulating directly on the bottom contact with a fiber using a pulse width of 500 ms. Output intensity was 120 μW. (**d**) Magnification of the stimulation artifact showing the bandpass filtered signal (black) and the raw signal (grey).

We designed the MEOA so the light originates from the same plane as the recording electrodes (i.e. the light beam is sent nearly to perpendicular to the substrate plane and away from the electrode). In addition to this, the emitted light beam is directional and confined, since the light output is coming out with an angle of 20° with respect to the normal (see Fig. 2), and diverges with an angle lower than 6°. Therefore, the TiN electrodes are not directly exposed to light. To prove that light stimulation did not cause any interference, we recorded from 2 electrodes in neurobasal medium solution while sending light through a waveguide towards the optrode located in between these two electrodes. Figure 4a shows the bandpass filtered data traces of the two electrodes recorded during an optrode activation. The light output power intensity was set to 5 μW, which was the same power intensity used to stimulate the neurons. No recording artifact was noticeable above the baseline noise level. Additionally, even when setting the output to a significantly higher light output power intensity (120 μW), no artifact was recorded (see Fig. 4b). On the contrary, when positioning an optical fiber directly above the TiN recording contact and illuminating it with the same light power (120 μW), a discernible artifact can be observed (see Fig. 4c,d).

### Optical neuromodulation of single cell activity

Precise neuromodulation requires fine control over neuronal excitability. In order to confirm that the combined electrical/optical array allows for localized stimulation of neurons *in vitro*, we cultured neurons on top of the MEOA. We then transduced the cultures with an AAV2/7-ChR2 vector and imaged them subsequently for identification of opsin expression prior to optical stimulation. Finally, to elicit time-locked spikes from neuronal soma’s and/or axons, we stimulated through one visually selected optrode while recording from neighboring electrodes.

The transduction rate was 51.8 ± 13.4% (5 cultures) as determined from cell counting of Alexa-647 (NeuN) and mCherry (ChR2) positive cells. This was more than sufficient to encounter transduced neurons on top of the optrodes to perform the necessary experiments.

**Figure 5:**
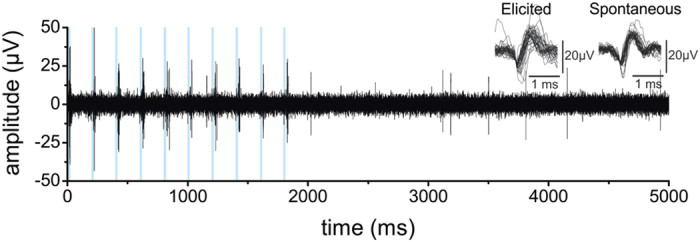
Example of single unit recordings under light modulation. Clear time-locked spikes were evoked by the pulse train (10 pulses at 5 Hz and pulse width of 20 ms). Spike waveforms are plotted as inset and were similar to the spontaneously fired spikes. The blue lines indicate light ON.

**Figure 6:**
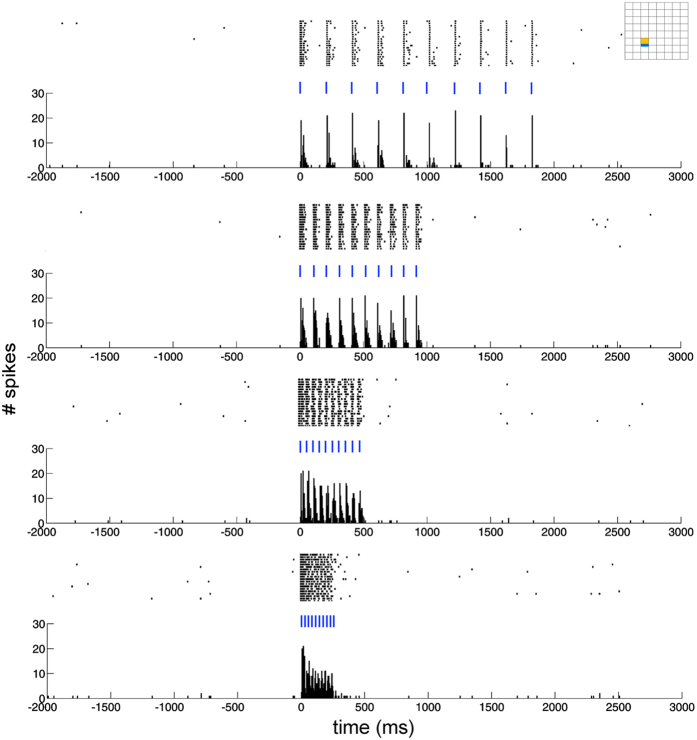
Rasterplots and peri-stimulus time histograms (PSTH) of time-locked elicited spikes after stimulation at different frequencies (5, 10, 20 and 40 Hz) with a pulse width of 5 ms. The blue lines indicate light ON. Inset on top right shows the location of the recorded contact (orange) and stimulated optrode (blue) on the MEOA. Bin size of PSTH’s was 5 ms.

When stimulating through one optrode and recording from the closest electrode, we could reliably elicit time-locked spikes at various frequencies and pulse widths. Figure 5 shows a filtered data trace during stimulation with 10 pulses of 20 ms repeated with a frequency of 5 Hz. The evoked spikes did not differ in shape from the spontaneous spikes fired by the neurons (Fig. 5, inset). As expected, preliminary data suggests the firing frequency increases and the spike latency decreases with higher power densities (see Supplementary Fig. S1). When applying pulse trains at different frequencies (5, 10, 20 and 40 Hz) using a pulse width of 5 ms, we noticed clear time-locking of the neuronal response to the stimulation pulses. Only at the highest frequency (40 Hz) the spike fidelity dropped slightly (see Fig. 6).

Recording from non-transduced neurons did not elicit any neuronal response, indicating that the illumination alone does not elicit a response (See Supplementary Fig. S2). Also, when deliberately misaligning the laser diode above the input grating in a way that no light can couple into the waveguides, the activation of the laser diode did not elicit a neuronal response from transduced neurons, indicating that the presence or operation of the laser diode itself close to the chip did not induce any electrical artifacts or stray light onto the MEOA (see Supplementary Fig. S3).

### Spatial and temporal mapping of network activity

Above-threshold (electrical or optical) stimulation causes depolarization and action potential (AP) generation in neurons. Those APs then propagate to neighboring cells through synaptic connections. A local stimulus will therefore thus propagate from one or more neurons throughout the neuronal network. This propagation can be monitored *in vitro* by fluorescent^25^ or electrical methods^26^. To demonstrate the MEOA’s ability to provide a full spatial mapping of synaptic network activity, we employed localized stimulation through a single optrode, while recording from all neighboring electrodes.

**Figure 7:**
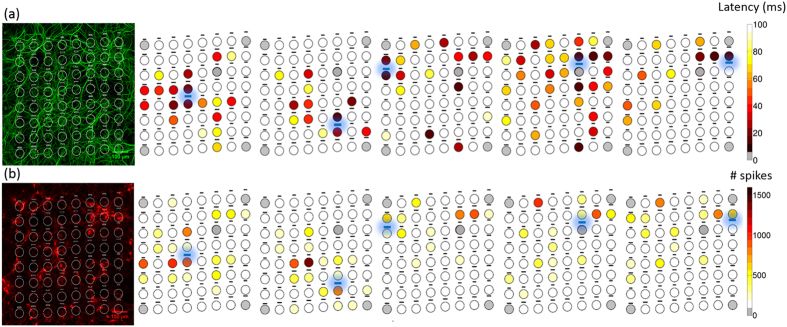
Spatially and temporally resolved neuronal activity from hippocampal neurons growing on top of the MEOA. Left: Confocal image of the neuronal dendrites (MAP2, top) and transduced neurons (mCherry, bottom) to depict the distribution of the neurons on the MEOA. Brightness and contrast are enhanced for visualization purposes. Right: Mapping of the latency to the first elicited spike (**a**) or the total spiking activity (**b**) during the stimulation ON period over 50 stimulation trials (0.2 Hz, 500 ms pulse train) show a clear temporal and spatial correlation to the stimulated optrode (colored in blue). Grey electrodes indicate sites that were not available for recording with the used system or broken contact.

We stimulated the neurons/axons growing atop of the optrode for a period of 500 ms every 5 seconds and recorded the neural response from all contacts of the MEOA. Data traces of the neural response were then converged into heat maps to present patterns of modulated single-unit activity (see Fig. 7b). In general, neurons recorded close to the optrode showed significant stronger activation (spikes/s) than neurons positioned further away (Spearman correlation coefficient R = -0.25, p< 0.0001, n = 258 active electrodes from 4 cultures). To demonstrate the temporal resolution of optical stimulation, we calculated the mean latency of the first spike on every electrode. Figure 7a shows a two dimensional map of the latencies, averaged over 50 trials. Again, the latencies of the activated neurons recorded close to the stimulated optrode were significantly smaller than those of the activated neurons further away (Spearman correlation coefficient R = 0.2, p = 0.002, n = 228 active electrodes from 4 cultures).

**Figure 8:**
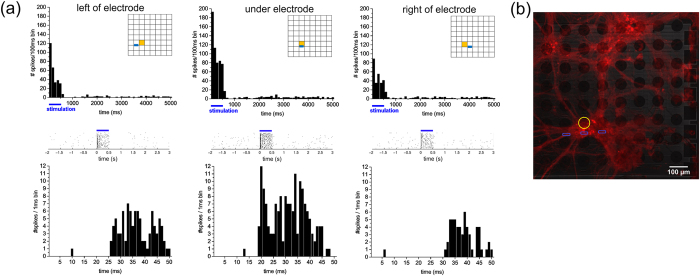
(**a**) Peri-stimulus time histograms (PSTH) (top), rasterplots (middle) and latency histograms (bottom) of elicited spikes from the same contact (see insets, in orange) when stimulating sequentially through three neighboring optrodes (see insets, in blue). Notice the stronger activation and shorter latency when stimulating through the middle and closest optrode compared to the optrodes one position left and right. (**b**) Confocal image of the transduced culture (mCherry) with the location of the recorded contact (in yellow) and the three stimulated optrodes (in blue).

Another representation of the spatial and temporal resolution of the device is shown in Fig. 8a. Here, it shows the peri-stimulus time histogram, rasterplots and latency histograms of elicited spikes recorded on one contact (see insets, in orange) when stimulating through three different neighboring optrodes (see insets, in blue). Stronger activation and shorter latency could be seen when stimulating through the closest optrode right under the electrode, compared to stimulation through the optrode one position to the left and right (Fig. 8b). Notice also that, even from this densely clustered culture, the MEOA is able to record different spatial and temporal activation patterns, as shown in the confocal image (see Fig. 8b) of the transduced culture.

**Figure 9:**
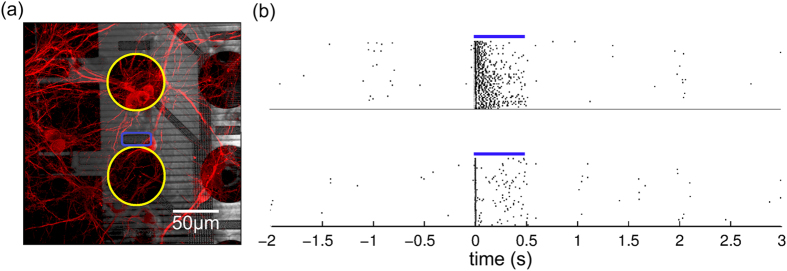
(**a**) Confocal image of part of the chip with transduced neurons and (**b**) rasterplots of the neuronal activation of two depicted contacts (in yellow), when stimulating through the optrode (in blue). Blue lines indicate light ON. The smaller latency of the measured activity on the bottom contact suggests recording of direct activation of the neuron on top of the optrode. The larger latency of the measured activity on the top contact suggests indirect (synaptic) activation of the neuron(s) on this contact.

One last example that suggests single-cell resolution of optical stimulation with this device is depicted in Fig. 9. From the recorded activity of the two contacts, that have each one or more transduced neurons on top, we could distinguish different activation patterns: the activity recorded on the bottom contact likely comes from the neuron that is positioned on the stimulated optrode, and has a smaller mean latency to the first spike (10.40 ms) than the activity recorded from the top electrode (14.47 ms). This latter activity is likely coming from the multiple units located on this electrode, which might be indirectly activated.

## Discussion

Electrical stimulation and recording of *in vitro* neurons are at present often performed using multi-electrode array (MEA) technologies. These devices are widely used in research involving synaptic plasticity^27^, visual perception^28^, and dynamics of neural networks^29^. Despite the fact that MEA’s have shown to be successful for high-resolution and non-invasive recording of neural activity^30^,^31^, simultaneous and targeted stimulation using electrodes *in vitro* is not straightforward. Some limitations fundamental to electrical stimulation are: (a) the cellular activity can only be activated, not inhibited; (b) electrical stimuli are non-specific to particular cell types; (c) it is not straightforward to limit an electrical field to a particular cell of interest. Moreover, the stimulation pulse might saturate the recording amplifier, which causes a ‘dead’ time between the pulse and the start of the recording.

Optical stimulation of cells is being increasingly used since the development of optogenetics. One of the main advantages of this technique compared to electrical stimulation is the cell type specific stimulation, enabling targeting of particular neural subtypes. For *in vitro* applications, light can be easily delivered to the sample using common light sources such as lasers^18^, LEDs^32^,^33^, or microscope-based solutions^34^. However, these setups are typically very bulky and expensive. Moreover, they are often restricted to the use of a single optical output, and the light is, except for the laser-based solutions, often not confined. Finally, even though regular (electrical) stimulation artifacts are avoided when using optogenetic techniques, these devices can still suffer from light-induced artifacts that disturb the recording^20^.

Various other systems for combined electrical recording and optical stimulation have been reported^7^,^8^,^9^,^10^,^11^. Nevertheless, the presented novel MEOA device offers important additional features. First, all of the 64 electrodes have adjacent optrodes. We introduced the light into the chip using a miniature diode laser, which was located at the edge of the chip, far away from the ‘active area’. This simplifies light coupling and prevents any stray light into the center of the array. Using embedded waveguides to transport the light also prevents undesirable heating of the sample. The small optrodes, made in CMOS compatible SiN technology, allowed for targeted stimulation in selected spots of the cellular network. Indeed, we showed that the cells were stimulated in a very confined area around the optrode, which allowed for single cell activation. This was further demonstrated through generation of clear temporal and spatial activation patterns within the neural network. The MEOA is thus an interesting tool to dissect neuronal networks in detail.

Next to the powerful single-cell resolution and precise network analysis capabilities, the MEOA has the benefit to be fabricated in a fully CMOS compatible fabrication process. This is an efficient wafer-level process yielding a large quantity of identical high-quality chips. Also, we embedded both optical and electrical components in a single process flow which increases functionality of the complete device drastically. The same technology can be employed for *in vivo* implementation, as *in vivo* optogenetics is a powerful tool to unravel neural circuits in the brain. However, some additional requirements to the system to achieve this application are necessary, e.g. a smaller size, both in term of width and thickness, of the *in vivo* probes compared to the large *in vitro* chip, and a small and light-weight package to be able to mount such a device on the animal’s head. Taken together, this novel approach could serve different applications for optogenetics and enable scientific breakthroughs in the field of neuroscience and beyond.

## Materials and Methods

### MEOA fabrication

The MEOA device has been monolithically fabricated on a 200 mm silicon wafer (see Fig. 3) using a back-end CMOS compatible process. For this, we merged two different modules, one for SiN waveguides^35^ and the other for TiN electrode array technology^30^.

### *In vitro* experiments

#### Hippocampal culture and transduction

Animals were handled in accordance with international (EU Directive 86/609/EEC) and national laws governing the protection of animals used for experimental purposes, minimizing distress during procedures. The use of animals and procedures was approved by the Ethical Committee for Animal Welfare (ECD, Ethische commissie Dierenwelzijn) of KULeuven and Imec. Rat (Wistar, Janvier) embryonic hippocampal neurons were prepared as described elsewhere^36^ and plated at a density of 50,000 to 100,000 cells per cm^2^ on poly-L-lysine (PLL, P2636, Sigma-Aldrich, 0.5 mg/mL in borate buffer) followed by 10 μg/ml laminin (L2020, Sigma-Aldrich) coated substrates. Cultures were grown in neurobasal medium supplemented with B27 and 10% fetal bovine serum for the first day *in vitro*, and thereafter in neurobasal medium with B27 at 37 °C and 5% CO_2_. The neurons were transduced at 2 days *in vitro* (DIV) with the vector AAV2/7 CaMKIIa-0,4-intron-ChR2-L132C/T159C-mCherry^37^ (20 μl in 750 μl medium, titer 2.3 × 10^12^ GC/ml) produced by the Leuven Viral Vector Core, and left to incubate for 48 h before refreshing medium.

#### Extracellular recording and stimulation

The recording and stimulation sessions were performed at 14-21 DIV. To activate the desired optrode, a miniature laser diode (Osram, PL450B) was placed on top of the corresponding input grating using a motorized micromanipulator. The laser diode was powered by a high precision AC and DC current source (Keithley, 6221) which was modulated by the STG2000 stimulator (Multichannel Systems, GmbH).

Recordings were obtained with the USB-ME32-FAI-System or the MEA1060-Inv-BC system (Multichannel Systems, GmbH) at 25 kHz (gain of 1000 and 1100, respectively) and band pass filtered between 300 Hz and 3 kHz. A trigger signal from the stimulator was used to synchronize the light pulses with the recordings.

Spikes were identified by threshold detection of 5 times the standard deviation of the noise level. Signal to noise ratios and baseline noise levels were calculated as reported in^38^ and^39^, respectively. Rasterplots, heat maps and peri-stimulus time histograms (PSTH’s) were obtained with custom-made Matlab scripts and Origin 8.0 software.

PSTH’s were calculated with a bin size of 5 or 100 ms, latency histograms with a bin size of 1 ms. The number of spikes was defined as the spike counts over 20 to 50 trials divided by the bin size. The heat maps were calculated using the total amount of spikes during the stimulation ON period over 50 trials. Correlations (non-parametric Spearman Rank R) were made between the distance of the recording electrode to the stimulated optrode and the total amount of spikes during the stimulation ON period or the mean latency to the first spike. The Wilcoxon matched pairs test was used to compared the impedance values of the electrodes before use and after 5 times re-use in culture. Significance was determined at *p <* 0.05.

The impedance at 1 kHz of a selection of electrodes was measured with the NanoZ impedance meter (Multi-Channel Systems GmbH) in phosphate buffered saline at pH 7.4 before initial use and after 5 times re-use in culture. In between cultures, the MEOA’s were cleaned overnight with a 1% Tergazyme® solution (Sigma-Aldrich) and rinsed with DI water and isopropylalcohol. Optical microscopy was used to confirm the surface cleanliness.

#### Fluorescence imaging

Neurons were fixed in paraformaldehyde (4% PFA and 4% Sucrose in PBS) at 37 °C for 15 min and permeabilized for 5 min in 0.1% Triton X-100/PBS, blocked for 20 min in 20% goat serum in PBS. They were incubated with the primary antibodies MAP2 (1:200, Abcam) and NeuN (1:200, Millipore) in PBS for 1 h at room temperature or at 4 °C overnight. Goat anti-Chicken IgY (H+L) secondary Antibody, AlexaFluor® 647 (1:500, Life Technology) and goat anti-mouse AlexaFluor® 488 (1:500, Life technology) was diluted in PBS and incubated for 4 h at room temperature. After washing with PBS, nuclei were visualized using the DAPI compound (Invitrogen). Images were taken using a Zeiss Laser Scanning Microscope (LSM 780) and a 10× or 40× immersion objective at a sub-saturating exposure time. The ImageJ software was used to calculate the transduction rate as the relative percentage M-Cherry positive cells (transduced cells) to the NeuN positive nuclei.

### Optical characterization

The optrodes were activated as explained above using a miniature laser diode and a micromanipulator. With a second micromanipulator, an optical fiber (Thorlabs, FG105UCA) was placed on top of the optrode to collect the emitted light. The intensity of the captured light was measured with an optical power meter and a photosensor (Thorlabs, PM200 and S151C, respectively).

## Additional Information

**How to cite this article**: Welkenhuysen, M. *et al*. An integrated multi-electrode-optrode array for *in vitro* optogenetics. *Sci. Rep.* **6**, 20353; doi: 10.1038/srep20353 (2016).

## Supplementary Material

Supplementary Information

## References

[b1] Benabid A. L., Chabardes S., Mitrofanis J. & Pollak P. Deep brain stimulation of the subthalamic nucleus for the treatment of Parkinson’s disease. Lancet. Neurol. 8, 67–81 (2009). 19081516 10.1016/S1474-4422(08)70291-6

[b2] Nuttin B., Cosyns P., Demeulemeester H., Gybels J. & Meyerson B. Electrical stimulation in anterior limbs of internal capsules in patients with obsessive-compulsive disorder. Lancet 354, 1526 (1999). 10551504 10.1016/S0140-6736(99)02376-4

[b3] Deisseroth K. Optogenetics. Nat. Methods 8, 26–9 (2011). 21191368 10.1038/nmeth.f.324 PMC6814250

[b4] Boyden E. S., Zhang F., Bamberg E., Nagel G. & Deisseroth K. Millisecond-timescale, genetically targeted optical control of neural activity. Nat. Neurosci. 8, 1263–8 (2005). 16116447 10.1038/nn1525

[b5] Nagel G. . Channelrhodopsin-2, a directly light-gated cation-selective membrane channel. Proc. Natl. Acad. Sci. USA 100, 13940–5 (2003). 14615590 10.1073/pnas.1936192100 PMC283525

[b6] Schobert B. & Lanyi J. K. Halorhodopsin is a light-driven chloride pump. J. Biol. Chem. 257, 10306–13 (1982). 7107607

[b7] Zorzos A. N., Scholvin J., Boyden E. S. & Fonstad C. G. Three-dimensional multiwaveguide probe array for light delivery to distributed brain circuits. Opt. Lett. 37, 4841–3 (2012). 23202064 10.1364/OL.37.004841 PMC3572236

[b8] Aravanis A. M. . An optical neural interface: *in vivo* control of rodent motor cortex with integrated fiberoptic and optogenetic technology. J. Neural Eng. 4, S143–56 (2007). 17873414 10.1088/1741-2560/4/3/S02

[b9] Im M., Cho I.-J., Wu F., Wise K. D. & Yoon E. Neural probes integrated with optical mixer/splitter waveguides and multiple stimulation sites. In *2011 IEEE 24th International Conference on Micro Electro Mechanical Systems* 1051–1054 (IEEE, 2011). 10.1109/MEMSYS.2011.5734609

[b10] Zhang J. . Integrated device for optical stimulation and spatiotemporal electrical recording of neural activity in light-sensitized brain tissue. J. Neural Eng. 6, 055007 (2009). 19721185 10.1088/1741-2560/6/5/055007 PMC2921864

[b11] Schwaerzle M., Seidl K., Schwarz U. T., Paul O. & Ruther P. Ultracompact optrode with integrated laser diode chips and SU-8 waveguides for optogenetic applications. In *2013 IEEE 26th International Conference on Micro Electro Mechanical Systems (MEMS)* 1029–1032 (IEEE, 2013). 10.1109/MEMSYS.2013.6474424

[b12] Schwaerzle M., Pothof F., Paul O. & Ruther P. High-resolution neural depth probe with integrated 460 NM light emitting diode for optogenetic applications. In *2015 Transducers - 2015 18th International Conference on Solid-State Sensors, Actuators and Microsystems (TRANSDUCERS)* 1774–1777 (IEEE, 2015). 10.1109/TRANSDUCERS.2015.7181290

[b13] Anikeeva P. . Optetrode: a multichannel readout for optogenetic control in freely moving mice. Nat. Neurosci. 15, 163–70 (2012). 22138641 10.1038/nn.2992 PMC4164695

[b14] Cho I.-J., Baac H. W. & Yoon E. A 16-SITE NEURAL PROBE INTEGRATED WITH A WAVEGUIDE FOR OPTICAL STIMULATION Il-Joo Cho, Hyoung Won Baac and Euisik Yoon Department of Electrical Engineering and Computer Science. 995–998 (2010).

[b15] Rossi M. A. . A wirelessly controlled implantable LED system for deep brain optogenetic stimulation. Front. Integr. Neurosci. 9, 8 (2015). 25713516 10.3389/fnint.2015.00008 PMC4322607

[b16] Grossman N. . Multi-site optical excitation using ChR2 and micro-LED array. J. Neural Eng. 7, 16004 (2010). 20075504 10.1088/1741-2560/7/1/016004

[b17] Kim T. . Injectable, cellular-scale optoelectronics with applications for wireless optogenetics. Science 340, 211–6 (2013). 23580530 10.1126/science.1232437 PMC3769938

[b18] Cardin J. A. . Targeted optogenetic stimulation and recording of neurons *in vivo* using cell-type-specific expression of Channelrhodopsin-2. Nat. Protoc. 5, 247–54 (2010). 20134425 10.1038/nprot.2009.228 PMC3655719

[b19] Wang J. . Integrated device for combined optical neuromodulation and electrical recording for chronic *in vivo* applications. J. Neural Eng. 9, 016001 (2012). 22156042 10.1088/1741-2560/9/1/016001

[b20] Yakushenko A. . On-chip optical stimulation and electrical recording from cells. J. Biomed. Opt. 18, 111402 (2013). 23788259 10.1117/1.JBO.18.11.111402

[b21] Tamir T. & Peng S. T. Analysis and design of grating couplers. Appl. Phys. 14, 235–254 (1977).

[b22] Boyden E. S., Zhang F., Bamberg E., Nagel G. & Deisseroth K. Millisecond-timescale, genetically targeted optical control of neural activity. Nat. Neurosci. 8, 1263–8 (2005). 16116447 10.1038/nn1525

[b23] Massiani Y., Medjahed A., Gravier P., Argème L. & Fedrizzi L. Electrochemical study of titanium nitride films obtained by reactive sputtering. Thin Solid Films 191, 305–316 (1990).

[b24] Lewis N. S. Photoeffects at the Semiconductor/Liquid Interface. Annual Review of Materials Science (1984). at http://authors.library.caltech.edu/32298/

[b25] Sheridan G. K., Moeendarbary E., Pickering M., O’Connor J. J. & Murphy K. J. Theta-burst stimulation of hippocampal slices induces network-level calcium oscillations and activates analogous gene transcription to spatial learning. PLoS One 9, e100546 (2014). 24950243 10.1371/journal.pone.0100546 PMC4065069

[b26] Wagenaar D. A., Madhavan R., Pine J. & Potter S. M. Controlling bursting in cortical cultures with closed-loop multi-electrode stimulation. J. Neurosci. 25, 680–8 (2005). 15659605 10.1523/JNEUROSCI.4209-04.2005 PMC2663856

[b27] Lanté F., de Jésus Ferreira M.-C., Guiramand J., Récasens M. & Vignes M. Low-frequency stimulation induces a new form of LTP, metabotropic glutamate (mGlu5) receptor- and PKA-dependent, in the CA1 area of the rat hippocampus. Hippocampus 16, 345–60 (2006). 16302229 10.1002/hipo.20146

[b28] Cang J. . Development of precise maps in visual cortex requires patterned spontaneous activity in the retina. Neuron 48, 797–809 (2005). 16337917 10.1016/j.neuron.2005.09.015 PMC2562716

[b29] Hofmann F. & Bading H. Long term recordings with microelectrode arrays: studies of transcription-dependent neuronal plasticity and axonal regeneration. J. Physiol. Paris 99, 125–32 16442786 10.1016/j.jphysparis.2005.12.005

[b30] Huys R. . Single-cell recording and stimulation with a 16k micro-nail electrode array integrated on a 0.18 μm CMOS chip. Lab Chip 12, 1274–80 (2012). 22337001 10.1039/c2lc21037a

[b31] Braeken D. . Open-cell recording of action potentials using active electrode arrays. Lab Chip 12, 4397–402 (2012). 22930315 10.1039/c2lc40656j

[b32] Wang H. . Molecular determinants differentiating photocurrent properties of two channelrhodopsins from chlamydomonas. J. Biol. Chem. 284, 5685–96 (2009). 19103605 10.1074/jbc.M807632200

[b33] Lignani G. . Long-term optical stimulation of channelrhodopsin-expressing neurons to study network plasticity. Front. Mol. Neurosci. 6, 22 (2013). 23970852 10.3389/fnmol.2013.00022 PMC3747358

[b34] Tønnesen J. . Functional integration of grafted neural stem cell-derived dopaminergic neurons monitored by optogenetics in an *in vitro* Parkinson model. PLoS One 6, e17560 (2011). 21394212 10.1371/journal.pone.0017560 PMC3048875

[b35] Subramanian A. Z. . Low-Loss Singlemode PECVD Silicon Nitride Photonic Wire Waveguides for 532–900 nm Wavelength Window Fabricated Within a CMOS Pilot Line. IEEE Photonics J. 5, 2202809–2202809 (2013).

[b36] Goslin K. & Banker G. Experimental observations on the development of polarity by hippocampal neurons in culture. J. Cell Biol. 108, 1507–16 (1989). 2925793 10.1083/jcb.108.4.1507 PMC2115496

[b37] Pan Z.-H., Ganjawala T. H., Lu Q., Ivanova E. & Zhang Z. ChR2 mutants at L132 and T159 with improved operational light sensitivity for vision restoration. PLoS One 9, e98924 (2014). 24901492 10.1371/journal.pone.0098924 PMC4047080

[b38] Rutishauser U., Schuman E. M. & Mamelak A. N. Online detection and sorting of extracellularly recorded action potentials in human medial temporal lobe recordings, *in vivo*. J. Neurosci. Methods 154, 204–24 (2006). 16488479 10.1016/j.jneumeth.2005.12.033

[b39] Quiroga R. Q., Nadasdy Z. & Ben-Shaul Y. Unsupervised Spike Detection and Sorting with Wavelets and Superparamagnetic Clustering. Neural Comput. 16, 1661–1687 (2004). 15228749 10.1162/089976604774201631

