# Supplementary material for: An integrated multi-electrode-optrode array for in vitro optogenetics
Source: Sci Rep. 2016 Feb 2;6:20353. doi: 10.1038/srep20353 (PMC4735812; doi:10.1038/srep20353)
Supplement: Supplementary Information [file srep20353-s1.pdf]

Supplementary figures

An integrated multi-electrode-optrode array for in vitro optogenetics

Marleen Welkenhuysen, Luis Hoffman, Luo Zhengxiang, Anabel De Proft, Chris Van den Haute,

Veerle Baekelandt, Zeger Debyser, Georges Gielen, Robert Puers and Dries Braeken.

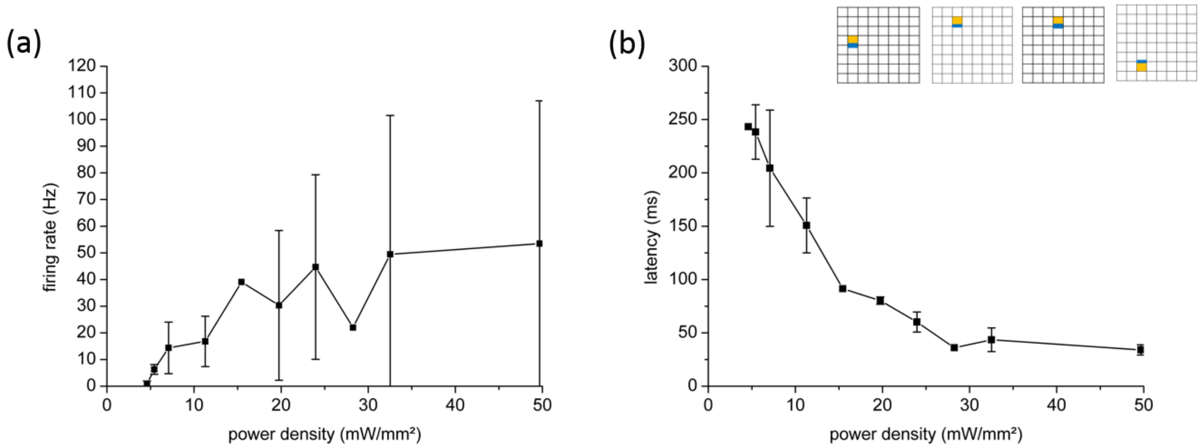

Figure S1: Mean ± standard deviation of the firing rate (a) and latency to the first spike (b) during the stimulation ON period (50 epochs of 500 ms at 0.2 Hz) in relation to the power density. Data suggests a stronger activation and smaller latency at higher emission power. Measurements taken from one contact on 4 different MEA's (see inset; recorded contacts in orange, stimulated optrodes in blue).

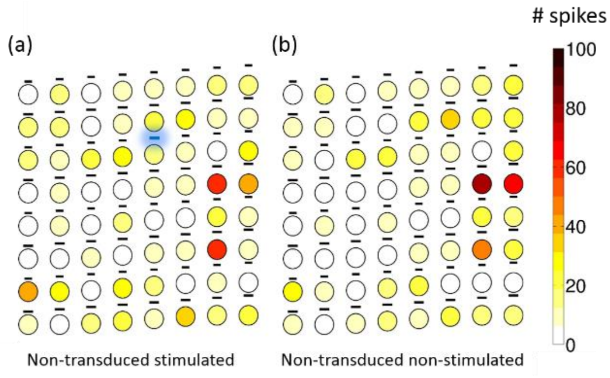

Figure S2: Spatially resolved neuronal activity from non-transduced hippocampal neurons growing on top of the MEOA. (a) Mapping of the total spiking activity during the stimulation ON period over 50 stimulation trials (0.2 Hz, 500 ms pulse train) shows no spatial correlation to the stimulated optrode (colored in blue). (b) Stimulation did not elicit a change in neuronal response from transduced neurons compared to baseline activity.

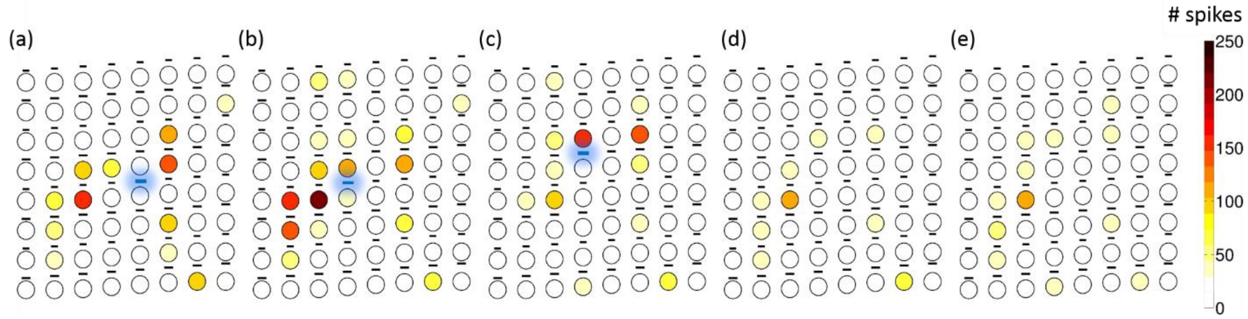

Figure S3: Spatially resolved neuronal activity from hippocampal neurons growing on top of the MEOA. (a-c) Mapping of the total spiking activity during the stimulation ON period over 50 stimulation trials (0.2 Hz, 500 ms pulse train) show a clear spatial correlation to the stimulated optrode (colored in blue). (d) Mapping of the total spiking activity when deliberately misaligning the laser diode above the input gratings. Stimulation did not elicit a change in neuronal response from transduced neurons compared to baseline activity (e).
